# General Limitations to Endophytic Entomopathogenic Fungi Use as Plant Growth Promoters, Pests and Pathogens Biocontrol Agents

**DOI:** 10.3390/plants10102119

**Published:** 2021-10-06

**Authors:** Bamisope Steve Bamisile, Junaid Ali Siddiqui, Komivi Senyo Akutse, Luis Carlos Ramos Aguila, Yijuan Xu

**Affiliations:** 1 Department of Entomology, South China Agricultural University, Guangzhou 510642, China; bamisopebamisile@gmail.com; 2 Plant Health Theme, International Centre of Insect Physiology and Ecology, Nairobi 00100, Kenya; kakutse@icipe.org; 3 State Key Laboratory of Ecological Pest Control for Fujian and Taiwan Crops, College of Plant Protection, Fujian Agriculture and Forestry University, Fuzhou 350002, China; luisramosaguila@gmail.com

**Keywords:** biological control, organic fertilizers, plant-fungal interactions, biotechnology, mutualism, plant nutrients

## Abstract

The multiple roles of fungal entomopathogens in host plants’ growth promotion, pest and pathogen management have drawn huge attention for investigation. Endophytic species are known to influence various activities of their associated host plants, and the endophyte-colonized plants have been demonstrated to gain huge benefits from these symbiotic associations. The potential application of fungal endophytes as alternative to inorganic fertilizers for crop improvement has often been proposed. Similarly, various strains of insect pathogenic fungi have been formulated for use as mycopesticides and have been suggested as long-term replacement for the synthetic pesticides that are commonly in use. The numerous concerns about the negative effects of synthetic chemical pesticides have also driven attention towards developing eco-friendly pest management techniques. However, several factors have been underlined to be militating the successful adoption of entomopathogenic fungi and fungal endophytes as plant promoting, pests and diseases control bio-agents. The difficulties in isolation and characterization of novel strains, negative effects of geographical location, vegetation type and human disturbance on fungal entomopathogens, are among the numerous setbacks that have been documented. Although, the latest advances in biotechnology and microbial studies have provided means of overcoming many of these problems. For instance, studies have suggested measures for mitigating the negative effects of biotic and abiotic stressors on entomopathogenic fungi in inundative application on the field, or when applied in the form of fungal endophytes. In spite of these efforts, more studies are needed to be done to achieve the goal of improving the overall effectiveness and increase in the level of acceptance of entomopathogenic fungi and their products as an integral part of the integrated pest management programs, as well as potential adoption as an alternative to inorganic fertilizers and pesticides.

## 1. Introduction

Entomopathogenic fungi (EPF) are complex species of insect pathogenic fungi that are popular for their ability to cause infections in their associated insect hosts. They directly penetrate the insect cuticle by invading the insect hemocoel through a contact mode of action [1]. Fungal entomopathogens have been employed as pest biocontrol agents in many countries for over 200 years [2]. In addition to insect pest control, their ability to colonize plants and exist as fungal endophytes is unique and adds a new dimension to their utilization as biological agents for crop management [3,4]. Fungal endophytes have been found existing in most of the plant species examined until now. This discovery has raised questions whether all plants maintain these endophytic associations with fungi [5], where plants from more than 100 families have so far been confirmed to harbor endophytic fungi [6]. Fungal endophytes have so far been reported in sorghum [7], wheat [8,9], corn [10,11], common bean [12,13,14], tomato [15,16], pumpkin [8], soybean [9], coffee [17], white jute [18], cotton [8,15,19,20], cacao [21,22,23], opium [24,25], cassava [26], banana [27,28] and in several other economically important crops [29]. Most fungal endophytes colonize the roots and aboveground tissues of their hosts systemically. Similarly, the ability has been found in many temperate grass species, and the association varies from pathogenic to mutualistic [6]. The mechanisms of endophytic fungi induced host defense against pests and pathogenic organisms have been explored many times [15,30,31,32,33]. One of the most commonly mentioned mechanisms is the ability of these fungal endophytes to synthesize bioactive compounds, which do not only build up plant resistance to pests and diseases, but could improve the rate of nutrients uptake, tolerance to abiotic and biotic stressors, and general plant growth improvement as well [34]. 

Several endophytic fungi-derived essential natural products have been synthesized for use in the medical field and for other commercial purposes. Products such as alkaloids, steroids, flavonoids, and terpenoids, etc., have been utilized in the discovery of new drugs and other medicinal products [35]. Considering these unique attributes of fungal endophytes, potential development as biopesticides for multipurpose utilization in integrated pest management programs has been mentioned. However, in-spite of the numerous importance and potentials of these fungal endophytes and their derivatives, the degree of efficiency, level of utilization as bio-agents for pests and pathogens, degree of successful adoption and general acceptance by users is still generally poor. The reason for the poor performance are arguably due to several of the challenges faced during isolation, characterization and subsequent utilization of EPF/fungal endophytes in the laboratory, green houses, as well as, on the larger scale, in field trials [36]. In the past couple of years, combating these numerous constraints have being the focus of several scientific studies [4,37]. The current article highlights the common limitations to EPF/fungal endophytes’ potential application as sustainable biological agents for plant growth promotion, insect pests, and disease causal organisms’ management. The possible solutions to the problems, various recent scientific developments and the future research prospects were also discussed. We are of the opinion that the general knowledge of these findings would help to improve the application, level of effectiveness, and overall adoption of EPF and fungal endophytes for pests and diseases management programs.

## 2. Specific Approaches for Entomopathogenic Fungi Application as Pest Biocontrol Agents

EPF exist as natural enemies of insect pests and regulate their populations in the ecosystem. Several strains of insect pathogenic fungi have been examined for their ability to cause mortality in the targeted insects and have thus been proposed for potential use as biological control agents. Since many decades ago, the traditional application of EPF as biological control agents involves continuous application of fungal conidia in the cropping system, usually in small to large quantities, with the sole aim of keeping pest populations at a level below the economic threshold [38]. In the recent years, EPF-centered biological control approaches still follow the same inundative spraying pattern, as well as, inoculative biological control strategy [39]. EPF can be applied for insect pest management using three broad biocontrol approaches, namely classical, augmentation and conservation biological controls. 

Under classical biological control, natural enemies are utilized to control exotic hosts that have established in a new location/environment where the natural enemies are naturally absent [40]. This approach generally provides a long-term sustainable and economical control of insect pests. Fungal entomopathogens boast a long history of application using this approach. As early as in the early 1900s, an entomopathogenic fungus *Entomophaga maimaiga* Hamber, Shimauzu and Soper (Entomophtorales: Entomophtoraceae) was isolated in Japan and released in the United States for the control of the larvae of the gypsy moth, *Lymantria dispar* Linnaeus (Lepidoptera: Lymantriidae) [38]. Following the same approach, another entomopathogenic fungus *Zoophthora radicans* (Brefeld) Batko (Zygomycetes: Entomophthorales), originally from Israel, was successfully introduced to Australia for the control of the spotted alfalfa aphid, *Therioaphis trifolii* (Monell) f. *maculate* (Homoptera: Drepanosiphidae) [38]. Furthermore, following successful introduction, fungal infection on the targeted hosts was reported to spread to other areas due to airborne movement of the conidia and human manipulation. For *E. maimaiga*, for instance, the fungus was reported covering the entire forests of northeastern USA, thereby suppressing *L. dispar* populations [41]. 

Augmentation involves the introduction of natural enemies into new areas where they are less active or too few in number to successfully minimize crop damage. Using this approach, fungal entomopathogens are commonly introduced using an inundative strategy or otherwise, using inoculation methods. In the latter approach, EPF are applied in small quantities with the expectation that the infection will become established in the pest populations and therefore maintain their level of activities below the economic threshold. An inundative strategy, on the other hand, involves continuous spraying of fungal spores, often in large quantities in the form of a mycopesticide [42]. The species of hyphomycete fungi that can easily be mass-produced and formulated as mycopesticides are the common inundative biocontrol agents. As inundative biocontrol agents, many isolates of hyphomycete fungi, namely; *Beauveria bassiana* (Balsamo) Vuillemin, *Metarhizium anisopliae* (Metschnikoff) Sorokin, *Lecanicillium lecanii* (Zimmerman) Viegas, etc., have been used for the control of a wide range of insect pest species in numerous countries, both in glasshouses and open fields. In many glasshouses in Europe, *L. lecanii* has been relatively successful against aphids and related insects [38]. Similarly, *M. anisopliae* var. *acridum* has recorded a promising level of success for the management of grasshoppers and locusts in Africa, while in North America, *B. bassiana* has widely been used and observed to be highly virulent against a wide range of economic insect pests [38]. 

The other approach that involves the reform of farming practices or habitat management to augment the activities of the entomopathogen populations is known as conservation [43]. To adopt this method, a proper examination of the existing indigenous natural enemies in the target area needs to be carried out. Thereafter, management practices that could help conserve or promote the activities of the natural enemies are adopted [44]. For EPF, management practices such as farmland irrigation to increase soil moisture, provision of overwintering sites of alternative hosts, and minimizing the rate of chemical insecticides application, have been confirmed to enhance fungal community richness [38]. 

In overall, EPF have a worldwide distribution, as different isolates from the genera *Metarhizium*, *Beauveria*, *Isaria* have often been isolated from different insect species and soil types. These fungal isolates have been targeted and recorded a substantial level of virulence against many insect pests of economic importance; grasshoppers, locusts, termites, spittlebugs and other hemipterans, noctuids, soil-borne insects (scarab species and curculionids), greenhouse pests (white flies, aphids, and thrips), as well as ticks, mosquitoes and even cockroaches [2,45]. Some species have a broader host range, while others are more restricted or host specific. For instance, the host range of *M. anisopliae* is more restricted than that of *B. bassiana*. Nevertheless, *M. anisopliae* has been reported against many insect orders including Coleoptera, Dermaptera, Diptera, Heteroptera, Homoptera, Hymenoptera, Isoptera, Lepidoptera, Orthoptera, Siphonaptera and Symphyla [46]. On the other hand, *Beauveria* has been recovered from several insect species, where over 707 species have been mentioned as insect hosts of *B. bassiana*, including 521 genera and 149 families of 15 orders, as well as an additional 13 Acarina host species from seven genera and six families. Despite the ubiquitous nature of the fungus, most isolates have a restricted host range. Similarly, *B. brongniartii* has been found to be highly specific for attacking European cockchafer, *Melolontha melolontha* Linneaus (Coleoptera, Scarabaeidae) and *M. hippocastani* Fabricius (Coleoptera, Scarabaeidae). However, the fungus also infect other insects, as many other fungal isolates from a distinct host insect are known to be highly virulent against other target pests. Tellingly, development of infection greatly depends on susceptibility of the target host, total population and the rate of successful transfer of fungal infection from infected hosts to the healthy ones. Certain insect species exhibit huge tolerance level to EPF treatment and have been found to be susceptible only to the fungal strains isolated from insects of the same species [2,38]. Although, some isolates have been found to exhibit host-insect preferences and are reported to be specific to certain insect order [47], in general, isolates from soil are suggested to be relatively highly virulent against specific insect pests [48]. 

## 3. Entomopathogenic Fungi Application in Plants

Aside from pest population suppression, the ability of EPF to colonize green plants and serve other functions as plant growth promoter, stress tolerance and water uptake enhancer, adds a new dimension to their potential utilization in plant management. Hence, the research into fungal endophytes is attracting more interest from scientists due to their roles in biocontrol, plant growth promotion and their potential application in the near future as a replacement to chemical pesticides and inorganic fertilizers [49]. Fungal endophytes serve as rich reservoir of biologically active substances, and their secondary products with novel structures and potential activities are of huge importance in agriculture, industries and in medicine [50,51]. They produce a broad range of secondary metabolites that are essential for use as insecticidal, antibacterial, and antifungal agents [52]. The mutual interactions between fungal endophytes and their colonized hosts have been found to be essential for protection against insect pests and disease-causing pathogens, as well as for host plant nutrition. The endophytes living in the host tissues draw benefits in terms of nutrition and protection, while also being directly involved in the nutrient uptake and protection of the host against biotic and abiotic stresses. Fungal endophytes generally influence the health, development, and growth of their hosts. Also, the plant community, ecosystem functioning and population dynamics are affected by the activities of the existing fungal endophytes [53].

## 4. The Constraints to Entomopathogenic Endophytic Fungi Application for Plant Improvement, Pests and Diseases Management

Microorganisms naturally colonize living plants, depending on their hosts for nutrition, shelter and protection from various environmental stressors [3,4]. The level of microbial colonization success is greatly influenced by the plant species [6]. The relationship between a healthy host and the fungal colonizer ranges from latent pathogenesis to mutualistic symbiosis. Aside from existing as endophytes, microorganisms could exist in other forms as epiphytes, or otherwise as latent pathogens. However, our focus is mainly on the fungal species that exist within the tissues of a living host without mediating any harmful effect, or manifesting any visible disease symptoms, therefore, generally termed as fungal endophytes [3,6]. In the past few decades, the pest management roles of these essential fungi have been well documented. Numerous studies have highlighted the existing relationships between fungi, their colonized plant hosts, soil and plant pathogens, as well as the additional benefits involving plant growth and health improvement [54]. Isolation, identification and application of microorganisms, especially the endophytic fungal species, is faced with a number of technical difficulties. For instance, the plant species and the manner of resources allocation in the whole plant influences the distribution of fungal endophytes, hence, the success level of novel fungal strains recovery is highly limited [55]. In addition, the underlying problems relating to the development of the insect pathogenic species as mycoinsecticides had previously been highlighted [56]. The major challenges encountered are related to the mass production, size and stability of propagules for storage and formulation [37,57,58]. The artificial inoculation method used for establishing endophytic EPF in the plant as endophyte has also been reported as a determining factor [17,29,59]. The ever-dynamic environmental factors, the persistent changes in the associated biological, physical and chemical conditions also greatly affect fungal endophytes abundance [36] (Table 1).

### 4.1. The Challenges in Isolation and Identification of Fungal Endophytes

Many species of fungi have been found occurring in the soil environment and have been suggested to have numerous ecological functions. For a long time until now, different isolation techniques have been utilized for successful recovery of several fungal strains from various soils and other biological samples [128]. Several, but not all of these fungal strains can be grown in vitro using various artificial media, where specific media have been developed to select for certain groups of microorganisms. For instance, Goettel and Inglis [71] selected a suitable specific media for isolating *Beauveria* spp. and *Metarhizium* spp. Hu and St Leger [72] also listed another selective medium for *Metarhizium* spp., otherwise known as Veens semi-selective medium, which was introduced by Veen and Ferron [129]. Veens medium contains the antibiotics chloramphenicol, the fungicides dodine and cyclohexamide [71]. Although, the medium can be successfully modified for isolation of other fungal species other than *M. anisopliae* [72], or for the recovery of soil applied conidia of *M. anisopliae* in the quest to examine the fungus persistence and vertical movement in the soil [130]. Another common method is the *Beauveria* sp. specific isolation media that was originally described by Strasser et al. [131]. The method has successfully been used in several other studies [132,133,134,135]. The culture-based techniques commonly used to check for fungal community diversity and composition are hugely limited to identifications based on morphological characteristics only. As a result, many fungal strains have been found to be unculturable, hence, measuring and identifying the endophyte community structure, composition and diversity has been a difficult task [36].

Aside from the aforementioned, several other growth media for the selective isolation of different EPF have been developed and are currently available [131]. However, the effectiveness of these media dependent methods is limited mainly by bacteria, which can inhibit the growth and recovery rate of targeted fungal entomopathogens. Although, this problem could be controlled with the help of broad-spectrum antibiotics such as tetracycline, streptomycin, or chloramphenicol [2,71]. The potential competition between the beneficial hypocrealean entomopathogenic fungi and the ubiquitous opportunistic saprophytes, however, remains a huge problem, as the targeted beneficial insect pathogenic fungal species grow relatively slower in comparison to the unwanted saprotrophic species. It has been suggested that, augmenting the media with reagents that could help prevent the opportunistic fungi from overgrowing the targeted species of interest could help solve the problem [66].

In addition to the semi and selective media, several other artificial isolation methods have been described, including those that are commonly used for fungi isolation from soils. The most common examples are the insect bait methods involving the use of larvae of the greater wax moth, *Galleria mellonella* Linnaeus (Lepidoptera: Pyralidae), instar larvae of European grapevine moth, *Lobesia botrana* Denis and Schiffermüller (Lepidoptera: Tortricidae), and mealworm larvae, *Tenebrio molitor* Linnaeus (Coleoptera: Tenebrionidae) [65,66,67,68,69,70]. Using the traditional insect bait methods, several researchers have evaluated different bait insects from different taxa. For instance, in the quest to isolate some distinct isolates, dipteran larvae such as larvae of *Delia floralis* Fallen (Anthomyiidae) could likely perform as a more suitable insect bait than the commonly used *G. mellonella* [136]. In this regard, the insect bait method is considered as a selective isolation method as only the insect pathogenic strains will generally be isolated using this techniques. However, the *Galleria* bait method would generally be more suitable, favorably considered and more sensitive than the media plating method [133], which is believed to have retained its popularity as the most commonly used method of fungi recovery.

The culture-based methods alone are not sufficient to explore the diversity and taxonomic composition of the fungal endophyte communities [6]. In recent times, modern molecular tools, such as the approaches based on restriction fragment length polymorphism (RFLP)—which allows individuals to be identified based on the unique patterns of restriction enzyme cutting in specific regions of DNA, together with other culture-independent techniques are now commonly used in place of the traditional selective media methods. 

In addition to the morphological characterization, molecular analysis can now be used for fungal identification and classification. Some of the common modern methods include polymerase chain reaction (PCR)-based analysis that is employed for amplification of DNA regions. Analysis of the ITS and large subunit (LSU) data are generally useful for minimizing the fungi identification related errors. Following amplification of DNA regions, purification procedures are initiated, including community fingerprinting or cloning techniques, which are relevant for analyzing endophyte communities [137,138,139].

Even though, very recently, researchers in advanced countries have found alternative ways to isolate and identify novel fungal strains, especially by employing various cultivation-independent techniques. However, across the various third world or developing countries of the world, there is every possibility that a larger percentage of fungal isolation and identification works still depend hugely on the traditional selective media procedures. It is also worthy of note that the analysis of plant DNA through next-generation sequencing is also faced with certain limitations. Some of the few challenges that have been identified include the inability to manage and conduct automation classification of large sequence datasets, as well as high diffusion of plant DNA against microbial DNA; hence, the separation of fungal metagenome for metagenomic analysis becomes a more difficult task to accomplish. The limited access to functional gene annotations in the gene library for genomes under study has also been mentioned among many other challenges [128,140].

### 4.2. Fungal Entomopathogens Irregular Distribution in the Soil

The climatic conditions, types of vegetation and soil, geographical location, activities of human and other biotic organisms are some of the factors that are well known to greatly influence the soil microbial communities [73,74]. The irregular localization or biodiversity of EPF in soils as a result of geographic and climatic conditions has been well highlighted in numerous studies. For instance, in a study conducted across Qinghai-Tibet Plateau and Gansu Corridor of China in 2016, it was reported that the likelihood of isolating novel strains of fungal entomopathogens is higher in the areas characterized as remote and less disturbed by human activities as against the regions with higher human disturbance [73]. Similarly, Cabrera-Mora et al. [69] isolated several species of EPF from various soils across different cropping systems in Mexico. They reported significant effects of crop types on fungal species diversity, while they also found substantial genetic diversity that could not be sufficiently explained using the geographical origin and the crop types. The authors concluded that different species of EPF could co-exist in the same soil ecosystem but in separate niches [69]. The influence of vegetation type on fungi localization has also been established in the study of Dong et al. [73], where several fungal strains were recovered from samples collected from areas with good vegetation cover, as against none that was isolated from soil samples collected from the desert. 

Soil serves as a reservoir and shelter for vast species of EPF, therefore, the soil is often targeted as potential source for isolation of novel and functional EPF strains [141,142]. Approximately 700 species belonging to over 90 genera have so far been suggested to have been isolated and identified [143]. Aside the rich diversity in soils, EPF are also widely distributed in several other agricultural and forest systems, such as green leaves, decaying trees, insect cadavers, etc. [144,145]. The insect pathogenic species, notably *Beauveria* and *Metarhizium* spp., which are of huge importance in agro-ecosystem and pest management programs, are the most popular and commonly isolated species. In this regard, several authors have collected data on the biology and ecology of several EPF species, including Samson et al. [146], Evans [147], and Eilenberg [148]. However, in spite of the diverse abundance, available data on their diversity and ecology is still limited, and the efforts to better explore the biology and ecology of fungi are greatly influenced by the aforementioned climatic and other edaphic factors. It has been suggested that a potential 1.5 million fungal species are present in the soil, while in contrast, around one tenth of these populations have been studied till date [149,150].

### 4.3. Level of Fungal Entomopathogens Virulence and Persistence on the Field

In addition to the negative effects of environmental conditions on distribution and abundance of entomopathogenic fungi, the level of virulence and persistence of these beneficial entomopathogens on the field are also greatly influenced by various negative environmental factors [73,151,152]. A large number of entomopathogenic fungal species are soil inhabiting, and several strains have been successfully isolated from various soils, however, soil moisture, temperature, pH, solar radiation, other soil-inhabiting microbes and organisms greatly influence the persistence, survival and level of effectiveness of EPF when used on the open field [123,124]. Temperature affects the rate of germination, growth and viability of fungal conidia in the laboratory, as well as on the field [121]. In addition, the storability of fungi is also affected by temperature. An example is the reduction in the viability of *M. anisopliae* dry conidia that was recorded in reaction to temperature change and exposure to light [153]. Humidity is another environmental factor that could as well influence both the efficacy and survival of fungal entomopathogens. High relative humidity (RH) is required for optimum germination of fungal conidia, as 100% RH has been found to be the most suitable for fungal spore germination [2,118]. 

In addition to the aforementioned environmental factors, solar ultraviolet radiation (UV) also plays an important role in determining the efficacy of fungal spores on the field, where UV-A and UV-B affect the survival and reduction of effectiveness on treated crops [124,126,127]. The vegetation type also plays a huge influence on the persistence of fungal entomopathogens on the field, as it affects the life cycle of the fungal spores. The spores, when in contact with soil-inhabiting insects and other suitable hosts under favorable conditions could initiate and complete their pathogenic process. At the completion of the infection process, newly formed spores are discharged from the cadavers and reenter the soil, where; depending on the soil conditions can be stored until the next infection process is initiated [154]. Tellingly, in response to negative environmental conditions or non-availability of favorable host, fungal entomopathogens are known to produce resting spores, which enhances a longer duration persistence in the soil [155]. However, this ability is only common with the entomophthoralean species, as this mechanism has not been fully defined in other species. For instance, in the aphid pathogen, *Pandora neoaphidis* (Remaudière & Hennebert) Humber, which is considered as the most important natural regulation agent of aphid colonies, the mechanism of hibernation in the fungus remains undiscovered. Nevertheless, the ability to form thick wall conidia in soil, or the formation of spherical hyphal bodies inside the insect cadavers, or through cycling in small populations of overwintering hosts, have all been suggested as possible modes of action [156,157,158]. Similarly, the formation of overwintering structures through the compression of hyphae (sclerotia), or otherwise, thick-walled resting spores (chlamydospores) are very common with Hyphomycete fungi. Notably in *Beauveria* spp., the infection rate in soil environment can be enhanced through radiation of modified hyphal strands outside the insect cadavers [63]. 

The ability of EPF to successfully colonize the inner tissues of their host plants and exist as fungal endophytes portends additional roles in nature and great importance in crop production [4]. In this regards, it has been suggested that EPF could be utilized in the form of fungal endophytes, as against the traditional method of application involving continuous spraying of fungal conidia on the plants. As this could help minimize the negative influence of unfavorable weather conditions on the fungal spores [29]. Fungal endophytes are known for their ability to exist in different environments and some have the capability to survive in extreme weather conditions [159]. Endophyte populations vary from species to species, as well as, from one plant to another. Within the same species, endophytic population might varies from region to region, while differences could also be recorded within the same region due to changes in climatic conditions. This implies that, the range of associated plants and fungi are altered as a result of climatic changes [160]. Plants may lose or gain endophytes due to temperature, rainfall, elevation, or latitude, as these are some of the known factors affecting the endophytic communities in plants [161]. 

A majority of the previously conducted studies on EPF/endophytes applications were laboratory or greenhouse studies, while only a few were field trials. In this vein, many of these studies have come short of addressing the problems relating to EPF/endophytes reduced efficacy on the field. Nevertheless, reports from some few available studies have indicated the success level of endophytic colonization in the treated plants was greatly reduced in natural field soils in comparison to sterile soils [13]. Another typical example is the mycopesticide Vertalec, which was formulated from *L. lecanii,* for the control of aphids. The product was reported to be very effective when applied in the glasshouse, where relative humidity could be modified to enhance optimum performance. However, utilization on open-field was limited as a result of its humidity requirements, hence, the market demand for Vertalec was relatively poor [119]. Several other field trials have demonstrated the need to prove the effectiveness, prospects and limitations of EPF in outdoor or uncontrolled conditions before they can be recommended for use commercially [20,25,75,76,77,78]. In order to develop a hugely successful biocontrol strategy, the need to further explore the ecological diversity and adaptability of fungi is imperative. As this would increase the understanding of the impact of environmental factors on the performance and persistence of the fungal spores.

### 4.4. Residual Effects on Predators, Parasitoids, and Other Non-Target Organisms

The major concerns regarding entomopathogenic fungi utilization in biological control strategies have been the potential side effects on natural enemies of insect pests, such as predators, parasitoids and other non-target organisms. In this light, various studies have been conducted in the laboratory, greenhouse, and on the field, to evaluate various strains of EPF for possible side effects against different non-target organisms, including pollinators, earthworms, spiders, honey bees, ants, other social insects, etc. [2,60,98,162,163,164,165]. Several of these reports have provided evidence of negative effects of EPF on honeybees [162,164,166,167], bumblebees [168], silkworm [169], and other natural enemies of insect pests [106,170,171]. 

For mycoinsecticide application on the field, non-target organisms are also a likely ultimate destination. Non-target organisms including plants, animals and microbes, represent a wide range category, which are non-targets, but are at high risk of exposure to mycoinsecticides. Among this category, the non-target insects are likely to be the most important destination, as many of the insect species are the primary hosts of the fungal spores from which the active ingredients of the mycoinsecticide were derived [172]. 

The soil is another main destination of mycoinsecticide drifting, thereby putting the soil-inhibiting organisms and beneficial soil microbes at huge risk. Fungal phages and mycotoxins could enter the soil via drifting pathways during application and dropping pathways from cadavers of targeted insect pests. Fungal spores can persist and survive in the soil for a long period [173,174,175,176]. The EPF species with a wide range of primary hosts such as *B. bassiana* and *M. anisopliae*, are the strains commonly implicated with spreading side effects on beneficial and non-target organisms [60]. Even though, there are suggestions that the negative effects are minimal and could therefore be ignored. For instance, Jaronski et al. [177] conducted various environmental assessment studies and concluded that the negative effects of *B. bassiana* on insect predators and parasitic wasps are relatively low and an acceptable risk. In addition, Nielsen et al. [171] also reported that the parasitic wasps (*Spalangia cameroni*) were moderately susceptible to *M. anisopliae*, while the total fecundity was similar to that of the untreated wasps. 

Similarly, Vestergaard et al. [64] also argued that the potential side effects of *B. bassiana* on beneficial insects, earthworms, honeybees, and plants could not be established based on the findings of the field studies conducted. However, there are still questions and safety risks related to the use of other fungal strains and mycoinsecticides, and these concerns have generated attention from many researchers and consumers. For *Isaria* based mycopesticides for instance, the main issues raised have been the environmental biosafety of their derived secondary metabolites and mycotoxins [172]. The comprehensive reports on the side effects of *B. bassiana*, *M. anisopliae*, and *B. brongniartii* on important insects and other non-target organisms, including earthworms, silkworms, bees and other pollinators, predators and parasitoids, have been presented by Danfa and Van der Valk [170], Zimmermann [2], Vestergaard et al. [64], Zimmermann [60], Goettel et al. [46], and many others [80,178].

### 4.5. Fungal Endophytes as Causative Agents of Livestock and Human Toxicosis

Endophytic fungi are responsible for the toxicity of several known poisonous plant species. The species generally referred to as grass–fungal endophytes are natural hosts and producers of multiple range of mycotoxins, alkaloids, and other physiologically active chemical compounds [91]. Majority of these biochemical compounds are notorious for causing problems for livestock, as they act as causal agents of livestock toxicoses and dangerous contaminants of rye crops for many centuries [6,91]. These secondary chemicals are responsible for more than 600 million dollars in losses due to dead livestock every year [92]. 

Some endophytic fungal species that play significant roles in host plant resistance against pest attacks and abiotic stresses have also been implicated in toxicity to grazing livestock. When not fatal, these biochemical compounds are responsible for reduced productivity in cattle and other livestock that are feeding on infected grasses [179]. Reduced nutritional value of infected plants can also minimize farm animal overall performances, which is due to reduction in feed intake by the animals [180,181]. The consequences of feeding animals with fungal infected plant matter is evident in the low pregnancy frequency and birthrate reduction that was recorded in horses and cattle [182]. The possibility of the various fungal endophytes derived toxic metabolites/toxins entering the plants has been highlighted. A typical example is beauvericin—derived from *Fusarium* spp, which is widespread in maize and other cereals. Although, findings of Moretti et al. [183] have established that, no visible disease symptoms due to beauvericin was observed in roots of barley, melon, tomato, and wheat plants, nevertheless, high toxicity was recorded in the protoplasts of all of the examined plants. 

Questions have often times been raised about the environmental safety of biological agents to users and animals alike. In fact, according to Zimmermann [2], the history of toxicology and pathological examinations dates back to 1967, when Schaerffenberg [79] conducted the first histological examinations on mammalian safety of *M. anisopliae*. The observations were carried out in adult white rats, involving injection, inhalation and feeding trials. The author reported that the rodents did not reveal any sign of toxicity or pathogenic reactions [79]. However, in contrast, in another toxicity assessment conducted by a biopesticides producing company known as Mycotech, two strains of *M. anisopliae* (*M. anisopliae* var. *anisopliae* and *M. anisopliae* var. *acridum*) were found to be extremely toxic to mice following their pulmonary (intranasal) observation [80]. Another fungus, *I. fumosorosea* was graded as a weak sensitizer, following records of low toxicities of acute oral, dermal, and inhalation to rats [81]. The authors reported that no eye irritation or dermal sensitization was observed on the treated rabbit skin [81,172]. However, the findings of Latch [93] somehow supported the claims of Schaerffenberg [79], where no abnormalities was recorded in the tissues of white mice and guinea pigs fed with *M. anisopliae* conidia for about 28 days. No weight loss or abnormal behavior was recorded following histological observations [93]. Another study also reported similar observations, where no evidence of ocular irritation or spore germination in animal tissues was found following spores injection or exposure of rats, mice and guinea pigs to *M. anisopliae* conidia [82]. Similarly, in another study, *M. anisopliae* has been confirmed to be safe for mammal following ingestion, inhalation, cutaneous and subcutaneous application in white mice and guinea pigs. It was observed from the anatomical and histopathological observations that the fungus is not pathogenic and non-toxic to the examined animals. Viable spores were discharged with the animal excrement, while subcutaneous observations showed that the conidia are only viable for one month in the body tissues [184]. Several other studies have reported similar findings in various animals following histopathological examinations [83,84,85,86]. However, another fungus, *B. bassiana*, has been implicated as the causal agent of mycotic keratitis in humans [87,88,89,90]. 

To this end, it has been revealed that certain toxic and beneficial alkaloids have been characterized with separate biochemical pathways, thereby, the development of strains with low animal toxicity while still maintaining high pest-tolerance properties is made possible.

### 4.6. Evidence of Endophytic Fungi Altering Host Defense System and Indirectly Promoting Pest Attack in Plant Hosts

The negative effects of endophytic fungi on the chemical composition of their host plants have been known by humans for centuries in terms of tissue poisoning and diseases [185,186]. The ability of endophytic EPF to offer protection to their hosts against herbivores has been exclusively discussed. However, it has been proven that, several endophytic symbioses do not confer protection against insect pests, as only few species associations serve as defense mutualisms [187]. Researchers have only managed to draw a thin line between mutualistic and pathogenic endophytes, as these lifestyles are hugely dependent on the interactions with several other species and the environmental conditions. It is therefore relevant to note that, the relationship that exist between endophytes and their hosts can be compared to a balanced antagonism, where, depending on the environmental conditions, the hosts could derive both negative and positive effects [188,189]. In this light, the environmental factors have a huge role to play in distinguishing between mutualistic and pathogenic endophytes [190,191]. The implication of the aforementioned conditions is that some fungal species that are known to be pathogenic to their hosts in the absence of primary pests of the host could become beneficial following intense pest attack, and vice versa. The common examples are fungal species that make their host cells sterile in place of their own nutrition and development [3,190]. The ability of some other species to provide protection for their hosts against their natural enemies at the expense of the host reproductive potential has also been mentioned. These species, majorly endomycorrhizae, render colonized hosts partially sterile while making preference for the fungal reproductive structures [3,192]. In addition, several species have been demonstrated to greatly influence nutrients and water uptake in their colonized host, however, plays little or no role in host defense against severe attack. These species are known to be unable to induce defensive chemicals in response to pest attack [193,194]. Whereas, some species can indirectly promote pest damage by making their hosts more susceptible to the herbivore [195]. According to Gange et al. [196], the overall effects on the host could be altered in a condition whereby multiple fungal strains are existing simultaneously in a single host. Although, the negative effects of several species of insects’ pathogenic fungi on their host plants are not very common, and in most cases, minimal. For instance, Zimmermann [2] argued that, there are no available reports of phytotoxic or phytopathogenic effects of *M. anisopliae* on the leaves or roots of the various hosts examined. Similarly, no negative reaction was recorded following treatment of strawberry and many other ornamental plants with different strains of *M. anisopliae* [2,197].

### 4.7. Epicuticular Waxes and Plant Volatiles Influencing Endophytic Entomopathogenic Fungi Activities in the Host Plants

The influence of soil type, rainwater, soil-inhabiting organisms and plant species, RH, temperature and other environmental conditions on the mobility, virulence, and persistence of fungal spores have been discussed in the previous sections. The aforementioned biotic and abiotic factors are widely regarded to be of huge importance due to the massive roles they play in EPF/fungal endophytes survival, fungal community diversity and composition [198,199]. However, the influence of the biochemical compounds contained in the host plants, such as the epicuticular waxes, root exudates and other plant volatiles cannot be overemphasized. For instance, it has been found that, some plant species with the help of their root and the root exudates exert negative effects on the activity of the fungus, *M. anisopliae* [2]. Similarly, the efficacy of *M. anisopliae* was reported to be reduced in cyclamen, where the fungus was evaluated in a greenhouse trial for the management of the black vine weevil, *Otiorhynchus sulcatus* Fabricius (Coleoptera: Curculionidae) [107]. In contrast, the positive influence of the epicuticular waxes and plant volatiles on the biocontrol activity of the same fungus, *M. anisopliae*, has also been reported. Certain plant species, most importantly the crucifers, such as Chinese cabbage (*Brassica rapa* var. *pekinensis*) and oilseed rape (*Brassica napus*), contain a blend of stimulatory and inhibitory compounds that are responsible for the modification of bioactivity of the fungus. The plant extracts were found to alter the rate of conidia germination and formulation on leaf extracts, which in turn increase the virulence of *M. anisopliae* [108]. On the other hand, another plant extract known as glucosinolates, contained in *B. napus* and other Brassicaceae, has been implicated in reduction of fungal spore germination rate, decrease in overall growth, and reduction in the ability of the fungus to infect insects. Following plant tissue damage or disorder, glucosinolates are hydrolysed to volatile isothiocyanates, which significantly inhibit *M. anisopliae* in vitro [109]. Another study has also revealed the inhibitory potentials of 2-phenylethyl isothiocyanate against *M. anisopliae* [110]. However, the plant extracts mediated-entomofungal pathogens inhibition has been established to be greatly influenced by the fungal species, plant type and the soil microcosm [110,200]. 

In addition, there are available reports on plant species influence on *B. bassiana* virulence and persistence against various insects [111,112,113,114]. Application of the fungus as biocontrol agent against chinch bugs, *Blissus leucopterus* Say (Heteroptera: Lygaeidae), reared on different plant species: barley, corn and sorghum, revealed significant differences in level of susceptibility across different plants. The bugs on barley showed less resistance to the fungus in comparison with those feeding on other plants [111]. Similarly, the silverleaf whitefly, *Bemisia argentifolii* Bellows and Perring (Homoptera: Aleyrodidae), reared on melon were significantly less resistance to *B. bassiana* when compared to the flies on cotton [113]. Similar dissimilarities in insects level of susceptibility to *B. bassiana* due to influence of plant species have also been found in the adults of tarnished plant bug, *Lygus lineolaris* Palisot de Beauvois (Hemiptera: Miridae) feeding on celery and lettuce plants [114], and in nymphs of glasshouse whitefly, *Trialeurodes vaporariorum* Westwood (Hemiptera: Aleyrodidae), reared on cucumber and tomato plants [112]. In the latter study, the nymphs feeding on tomato plants were found to be more resistant to *B. bassiana*. Here, it is believed that the inhibition of the fungus was due to the ability of *T. vaporariorum* nymphs to isolate the steroid alkaloid, tomatine, from the tomato plants. The detrimental effects of the glycoalkaloid on *B. bassiana* has been reported [112]. 

Scientific findings have also revealed that plant colonization efficiency is hugely dependent on the abundance, diversity, distribution and physiological status of the endophytes [201]. Furthermore, the variety or species of plant to be colonized has also been proposed as a very important factor to be critically considered when examining endophytic EPF for potential establishment as fungal endophytes in plant [9,202]. The plant hosts using the makeup of the root exudates determine the relevant microorganism interactions and symbiosis [203]. Therefore, the ability of the endophytic microbes to utilize the plant derived biochemical compounds as their energy sources greatly influence the interaction between the plants and the endophytic microorganisms [204].

## 5. Recent Research Advances and Future Research Needs

EPF are commonly used for pest management in cases where complete removal of the targeted pests is not required. The sole aim is to keep insect populations below the economic threshold. In this case, some minimal damage of crops is acceptable [38]. Different formulations and application mode of fungal entomopathogens have been employed for use in greenhouses and on open fields to combat pests attack, and have recorded varying degree of success against all kinds of insect pests with different feeding guilds, including aphids, locusts, thrips, grubs, moths, mites, whiteflies, etc. [76,205]. 

However, with the recent advances in microbial biotechnology, procedures are in place to enhance overall performance of EPF when used in the glasshouses or on the field [206,207]. Augmentation trials involving treatment of crop plants with fungal conidia in aqueous suspensions in combination with synthetic materials has been shown to be highly effective [38]. Several strains of EPF have been studied for potential application in combination with other chemical pesticides [54,208]. There are suggestions that the adoption of the combination would help improve resistance management strategies, as well as a reduction in ecosystem pollutions due to excessive use of inorganic insecticides [209]. In addition, there have been various field trials conducted to mollify the negative effects of ultra-violet radiation on fungal entomopathogens. For instance, skimmed milk was examined as a potential buffer against solar radiation when applying *B. brongniartii* in liquid culture on the field for the control of the European cockchafer, *M. melolontha* in barley. The fungus formed blastospores, which effectively caused a significant reduction in the populations of larvae and adult beetles over a long duration spanning to around nine years [38,210,211]. Many other studies have been conducted in the past to develop formulations with an improved UV stability [123,124,126]. 

Several of the emerging scientific studies are focus on exploring the ecology of fungal endophytes, their plant hosts, herbivores, and their multi-trophic interactions [212]. The molecular mechanisms related to EPF as biocontrol agents, and fungal endophytes induced host plant resistance are now the common goal of researches [213]. It is a general notion that a deeper understanding of the molecular mechanisms involved in plant hosts-endophyte relationships would enhance optimum utilization of EPF/fungal endophytes. Establishment of effective management strategies, through the evaluation of various inoculation methods for extended colonization of several fungal strains had also been suggested [29]. 

Meanwhile, gene modification procedures and development of RNA interference (RNAi) technology have made it possible to reconstruct fungal strains with the sole aim of improving the virulence and increase the overall performance [1]. The procedures involving the use of RNAi are now generally considered as novel techniques with the ability to control multiple ranges of agricultural pests [214,215,216]. However, despite the effectiveness of the techniques in the laboratory and in the glasshouses, the potential utilization on the field is hugely limited due to the challenges encountered in introducing exogenous dsRNA/siRNA into the target pests. In lieu of this major setback, most of the trials involving dsRNA/siRNA delivery methods have been limited to the laboratory, while only a few transgenic host plants have been successfully evaluated in field trials [216,217,218]. Similarly, detection and comparison of mRNA and protein expression levels of various genes related to plant host immunity and symbiotic association with endophytic fungi has helped to understand the many roles played by the associated genes. These roles include, but not limited to, signal transduction, cell death regulation, compound synthesis, nutrient acquisition or transport, nitrogen or iron metabolism, as well as, in many other plant–fungus interactions [219]. 

In the quest to identify many more culturable and non-culturable fungal endophytes present in the environment, genomic, proteomics, metagenomics and the rest of other omics techniques are now commonly used in the more advanced countries [36]. The advent of omics technology has also made the identification and classification of several uncharacterized taxa possible. In addition, isolated endophytes can easily be determined whether they are pathogenic, non-pathogenic, or beneficial endophytes [36,220]. The progress in microbial studies using molecular mechanisms has made it possible to draw a thick line of differentiation between beneficial and pathogenic fungi towards effective disease management and high-quality plant production. Scientific studies can now be effectively carried out to understand the molecular mechanisms involved in host plant responses to beneficial and pathogenic fungal interactions [219]. 

Although, there are several available reports on endophytic EPF, unfortunately, the data presented are still relatively limited and have shown inconsistencies under various conditions [221]. In addition, a larger percentage of these studies are biasedly and unfavorably focused on only few major important species, such as *Beauveria*, *Metarhizium* and *Lecanicillium* sp. It is imperative to isolate and identify several other novel fungal isolates with higher efficacy and potentials for controlling pests, pathogens, and even weeds. More research works should be focused on this direction. Al-Ani et al. [222] suggested the need to develop new fungal-derived secondary metabolites, while improving the efficacy of mycotoxins.

## 6. Conclusions

Fungal endophytes are now generally highly rated for their relevance in plant growth promotion, plant physiology balancing, plant nutrients restoration and phytoremediation. Due to their huge importance, endophytic fungi as well as other microorganisms, plants, animals and their by-products are now commonly targeted for the production of novel medicinal products and for other industrial applications. Although, at present, chemical pesticides and other synthetic compounds are still the commonly used measures for pests and plant management. However, with our focus on intensive pest management, optimum food production and ecosystem stability, in EPF and fungal endophytes, we have an alternative to chemical pesticides that is sustainable, cheap and ecofriendly. The various problems associated with EPF/fungal endophytes utilization, as we have discussed in this paper, are our major concerns. Nevertheless, the research into the biology and ecology of many fungal species could help overcome some of these problems, while a deeper understanding of the mechanisms they utilize in protecting their hosts from pests and pathogens is relevant for both effectiveness and commercialization. However, users are still faced with numerous setbacks with respect to large-scale production, marketing, appropriate formulation and application technology. In addition, some species of endophytic fungi have been identified to exist as opportunistic pathogens of plant, animal, or human; thereby raising concerns that their application as biological control agents of insect pests, for instance, might results in mild to severe illness or outbreak of other infections. In reality, the extent of acceptance, adoption and level of success of EPF is hugely dependent on the financial investment in research and development from the government, industries and other related agencies. In the light of this, it is very important for the government to step up and invest more in the biological control projects, especially in situations whereby significant commercial interests are limited or not readily available, in terms of development of classical, inoculative and conservation strategies.

## Figures and Tables

**Table 1:** Some of the biotic abiotic factors related with isolation, characterization and utilization of EPF/fungal endophytes for plant improvement, pests and diseases management.

		Specific Factors	Results/Observations	References
Fungal features	Epizootic potential	Spores germination	EPF require favorable environmental conditions to germinate, sporulate and cause infection in the targeted insect pests. The ability of EPF to establish disease epizootics is greatly influenced by various ecological conditions. In addition, successful spore germination can also be limited by certain cuticular lipids on the insects, including aldehydes, fatty acids, ketones, wax esters, and alcohols which may possess antimicrobial activity.	[2,38,60,61,62]
		Sporulation		
		Virulence		
		Persistence	EPF overall performance is limited due to lack of persistence and low rate of infection under challenging environmental conditions. Fungal persistence is hugely influenced by several abiotic and biotic factors, including soil moisture, temperature, soil microorganisms, soil-inhabiting insects and plants.	[2,60,61,63]
	Others	Fungi host range	Several fungal strains have a restricted host range. For instance, *B. bassiana*, despite the broad spectrum and prevalence in multiple insect orders, available reports have indicated that some *B. bassiana* strains are more restricted or host specific. The strains exhibit host-insect preferences and are specific to certain insect order. However, the host range of *M. anisopliae* is generally more restricted than that of *B. bassiana*.	[2,46,47,48,60,64]
		Isolation and characterization	Many fungal endophyte strains are unculturable. As a result, measuring and identifying the endophyte community structure and diversity has been a difficult task. Isolation of novel strains cannot rely on growth media based- or other traditional techniques only. Successful isolation of new fungal strains requires the application of molecular and other modern techniques.	[65,66,67,68,69,70,71,72]
		Spores localization/dispersal/mobility	The irregular localization or biodiversity of EPF in soils as a result of geographic and climatic conditions greatly influence fungal endophytes and EPF utilization. Soil moisture, soil type, soil organisms, and the plant roots influence the dispersal and mobility of fungal spores in the soil.	[60,69,73,74]
		Potentials for mass production	Until date, more than 170 fungal strains have been formulated as mycopesticides and are available for commercial use. However, mass production for commercial use is cost-intensive. In addition, significant decline in virulence due to changes in physiological and developmental process of fungi following repeated mass production of the same species on artificial medium has been reported. Additional underlying problems related to the development of EPF as mycoinsecticides have also been documented.	[2,20,25,56,60,61,75,76,77,78]
		Suitability for storage and formulation	EPF generally have short shelf life. Fungal conidia cannot be stored over a long duration. However, oil formulations can help protect fungal spores from the negative impact of harsh environmental conditions.	[61,62]
		Fungal toxicological and safety aspects	One of the major public concerns related to EPF application is the safety of users, other humans, animals, plants, natural enemies, pollinators and the general ecosystem. In addition, most biochemical compounds synthesized by fungal endophytes are notorious for causing problems for livestock.	[2,6,60,79,80,81,82,83,84,85,86,87,88,89,90,91,92,93]
		Compatibility with other pest and disease control techniques	The efficacy of EPF and fungal endophytes can be improved if applied in an integrative manner in combination with other biological, cultural or conventional measures. For instance, autodissemination strategies involving the combination of EPF and attractant traps or contamination devices has been effective. The compatibility of EPF with many other biological control agents, especially the associated natural enemies of targeted pests, such as predators and parasitoids would increase the overall level of performance.	[38,62,75,94,95,96,97,98]
Host features		Target host susceptibility	Infection development greatly depends on susceptibility of the target host, total population and the rate of successful transfer of fungal infection from infected hosts to the healthy ones. Certain insect species exhibit huge tolerance level to EPF treatment and have been found to be susceptible only to the fungal strains isolated from insects of the same species.	[2,38,99,100]
		Target host population	High population densities increase the chances of contact among individuals and improves pathogen transmission. Overpopulation increases the stress and fungal infection.	[101,102]
		Insect pests response to EPF volatile organic compounds	Some insect species including mole crickets, termites, and mites are able to detect and avoid soil or leaves treated with conidia of EPF belonging to the genera *Metarhizium* and *Beauveria*. Similarly, ants such as *Formica selysi* and *Lasius neglectus* workers can detect EPF and alter their behavior accordingly.	[103,104,105,106]
		Crop structure or constituents; Epicuticular waxes or plant volatiles	The host-plant chemistry influences fungal infection, by suppression or enhancing conidial germination and potential colonization. Plant species such as cyclamen, Chinese cabbage, oilseed rape, etc., are able to exert negative effects on the activity of fungi with the help of their root and the root exudates.	[2,60,107,108,109,110]
		Host-plant quality; effect of plant species	Plant species influence on *B. bassiana* virulence and persistence against various insects including silverleaf whitefly, tarnished plant bugs, and chinch bugs has been reported.	[60,111,112,113,114]
		Economic injury level	For inundative application, EPF are commonly applied following establishment of pests. The pest are to be present before the pathogen can be dispersed thus making preventive treatment difficult.	[38,61]
		Insect pests life stage	Some insects are able to evade fungal infection by molting. As a result, several EPF strains are unable to induce fungal disease in earlier larval stage.	[115,116,117]
		Density and spatial distribution of spore in relation to release or inoculation strategy	Most EPF strains are often slow acting and require application in very large quantity and thorough spray coverage.	[38,41,42,61]
Environmental factors	Abiotic factors	Relative humidity (RH)	Humidity affects the efficacy and survival of fungal pathogens. High humidity is required for optimum germination of fungal conidia. High RH promotes host cuticle penetration by conidia. 100% RH is the most suitable for fungal spore germination.	[2,60,62,118,119,120]
		Temperature	The rate of fungal conidia germination, growth and viability in the laboratory and on the field is affected by temperature. Most strains of insect-pathogenic fungi grow and sporulate at optimum temperature approximately 15–30 °C	[2,60,120,121,122]
		Soil moisture and PH	A large number of entomopathogenic fungal species are soil inhabiting, soil moisture and PH, in addition to soil temperature, in the presence of other soil-inhabiting microbes and organisms greatly influence the persistence, survival and level of effectiveness of EPF in the soil.	[123,124]
		Rain	Water applied to the soil through irrigation or during rainfall enhances migration/percolation of fungal spores into the soil. Water as dew or raindrops is responsible for dispersal of fungal conidia, while rainwater greatly influences the stability and efficacy of fungal spores on plants in the field.	[2,60,62,125]
		Irrigation		
		Dew drops		
		Solar ultraviolet radiation (UV)	Solar radiation determines the efficacy of fungal spores on the field. UV-A and UV-B affect the survival and reduce level of effectiveness on treated crops.	[60,123,124,126,127]
	Biotic factors	Microbial interaction	When EPF are treated in garden soils in greenhouses or on open fields, soil-inhabiting microbes and other soil-dwelling organisms greatly influence the persistence, survival and level of effectiveness of EPF.	[124,126,127]

## Data Availability

Not applicable.

## References

[1] Chen X., Li L., Hu Q., Zhang B., Wu W., Jin F., Jiang J. (2015). Expression of dsRNA in recombinant *Isaria fumosorosea* strain targets the *TLR7* gene in *Bemisia tabaci*. BMC Biotechnol..

[2] Zimmermann G. (2007). Review on safety of the entomopathogenic fungus *Metarhizium anisopliae*. Biocontrol Sci. Technol..

[3] Bamisile B.S., Dash C.K., Akutse K.S., Keppanan R., Wang L. (2018). Fungal Endophytes: Beyond Herbivore Management. Front. Microbiol..

[4] Vega F.E., Goettel M.S., Blackwell M., Chandler D., Jackson M.A., Keller S., Koike M., Maniania N.K., Monzon A., Ownley B.H. (2009). Fungal entomopathogens: New insights on their ecology. Fungal Ecol..

[5] Schardl C.L., Leuchtmann A., Spiering M.J. (2004). Symbioses of grasses with seedborne fungal endophytes. Annu. Rev. Plant Biol..

[6] Tadych M., White J.F., Moselio S., Schaechter M. (2009). Endophytic microbes. Encyclopedia of Microbiology.

[7] Tefera T., Vidal S. (2009). Effect of inoculation method and plant growth medium on endophytic colonization of sorghum by the entomopathogenic fungus *Beauveria bassiana*. BioControl.

[8] Gurulingappa P., Sword G.A., Murdoch G., McGee P.A. (2010). Colonization of crop plants by fungal entomopathogens and their effects on two insect pests when in planta. Biol. Control.

[9] Russo M.L., Pelizza S.A., Cabello M.N., Stenglein S.A., Scorsetti A.C. (2015). Endophytic colonisation of tobacco, corn, wheat and soybeans by the fungal entomopathogen *Beauveria bassiana* (Ascomycota, Hypocreales). Biocontrol Sci. Technol..

[10] Bing L.A., Lewis L.C. (1991). Suppression of *Ostrinia nubilalis* (Hübner) (Lepidoptera: Pyralidae) by endophytic *Beauveria bassiana* (Balsamo) Vuillemin. Environ. Entomol..

[11] Bing L.A., Lewis L.C. (1993). Occurrence of the entomopathogen *Beauveria bassiana* (Balsamo) Vuillemin in different tillage regimes and in *Zea mays* L. and virulence towards *Ostrinia nubilalis* (Hübner). Agric. Ecosyst. Environ..

[12] Akutse K.S., Maniania N., Fiaboe K., Van den Berg J., Ekesi S. (2013). Endophytic colonization of *Vicia faba* and *Phaseolus vulgaris* (Fabaceae) by fungal pathogens and their effects on the life-history parameters of *Liriomyza huidobrensis* (Diptera: Agromyzidae). Fungal Ecol..

[13] Parsa S., Ortiz V., Gómez-Jiménez M.I., Kramer M., Vega F.E. (2018). Root environment is a key determinant of fungal entomopathogen endophytism following seed treatment in the common bean, *Phaseolus*
*vulgaris*. Biol. Control.

[14] Parsa S., Ortiz V., Vega F.E. (2013). Establishing fungal entomopathogens as endophytes: Towards endophytic biological control. J. Vis. Exp..

[15] Ownley B.H., Griffin M.R., Klingeman W.E., Gwinn K.D., Moulton J.K., Pereira R.M. (2008). *Beauveria bassiana*: Endophytic colonization and plant disease control. J. Invertebr. Pathol..

[16] Qayyum M.A., Wakil W., Arif M.J., Sahi S.T., Dunlap C.A. (2015). Infection of *Helicoverpa armigera* by endophytic *Beauveria bassiana* colonizing tomato plants. Biol. Control.

[17] Posada F., Aime M.C., Peterson S.W., Rehner S.A., Vega F.E. (2007). Inoculation of coffee plants with the fungal entomopathogen *Beauveria bassiana* (Ascomycota: Hypocreales). Mycol. Res..

[18] Biswas C., Dey P., Satpathy S., Satya P., Mahapatra B. (2013). Endophytic colonization of white jute (*Corchorus capsularis*) plants by different *Beauveria bassiana* strains for managing stem weevil (*Apion corchori*). Phytoparasitica.

[19] Ownley B.H., Pereira R.M., Klingeman W.E., Quigley N.B., Leckie B.M., Lartey R.T., Caesar A.J. (2004). *Beauveria bassiana*, a dual purpose biocontrol organism, with activity against insect pests and plant pathogens. Emerging Concepts in Plant Health Management.

[20] Lopez D.C., Zhu-Salzman K., Ek-Ramos M.J., Sword G.A. (2014). The entomopathogenic fungal endophytes *Purpureocillium lilacinum* (formerly *Paecilomyces lilacinus*) and *Beauveria bassiana* negatively affect cotton aphid reproduction under both greenhouse and field conditions. PLoS ONE.

[21] Posada F., Vega F.E. (2005). Establishment of the fungal entomopathogen *Beauveria bassiana* (Ascomycota: Hypocreales) as an endophyte in cocoa seedlings (*Theobroma cacao*). Mycologia.

[22] Posada F.J., Chaves F.C., Gianfagna T.J., Pava-Ripoll M., Hebbar P. (2010). Establishment of the fungal entomopathogen *Beauveria bassiana* as an endophyte in cocoa pods (*Theobroma cacao* L.). Rev. UDCA Actual. Divulg. Científica.

[23] Rubini M.R., Silva-Ribeiro R.T., Pomella A.W., Maki C.S., Araújo W.L., dos Santos D.R., Azevedo J.L. (2005). Diversity of endophytic fungal community of cacao (*Theobroma cacao* L.) and biological control of *Crinipellis perniciosa*, causal agent of Witches’ Broom Disease. Int. J. Biol. Sci..

[24] Quesada-Moraga E., Landa B., Muñoz-Ledesma J., Jiménez-Diáz R., Santiago-Alvarez C. (2006). Endophytic colonisation of opium poppy, *Papaver somniferum*, by an entomopathogenic *Beauveria bassiana* strain. Mycopathologia.

[25] Quesada-Moraga E., Munoz-Ledesma F.J., Santiago-Alvarez C. (2009). Systemic Protection of *Papaver somniferum* L. Against *Iraella luteipes* (Hymenoptera: Cynipidae) by an Endophytic Strain of *Beauveria bassiana* (Ascomycota: Hypocreales). Environ. Entomol..

[26] Greenfield M., Gómez-Jiménez M.I., Ortiz V., Vega F.E., Kramer M., Parsa S. (2016). *Beauveria bassiana* and *Metarhizium anisopliae* endophytically colonize cassava roots following soil drench inoculation. Biol. Control.

[27] Pocasangre L., Sikora R., Vilich V., Schuster R. (2000). Survey of banana endophytic fungi from central America and screening for biological control of the burrowing nematode (*Radopholus similis*). InfoMusa.

[28] Akello J., Dubois T., Coyne D., Kyamanywa S. (2008). Endophytic *Beauveria bassiana* in banana (*Musa* spp.) reduces banana weevil (*Cosmopolites sordidus*) fitness and damage. Crop Prot..

[29] Bamisile B.S., Dash C.K., Akutse K.S., Keppanan R., Afolabi O.G., Hussain M., Qasim M., Wang L. (2018). Prospects of endophytic fungal entomopathogens as biocontrol and plant growth promoting agents: An insight on how artificial inoculation methods affect endophytic colonization of host plants. Microbiol. Res..

[30] Jaber L.R., Ownley B.H. (2018). Can we use entomopathogenic fungi as endophytes for dual biological control of insect pests and plant pathogens?. Biol. Control.

[31] Ownley B.H., Gwinn K.D., Vega F.E. (2010). Endophytic fungal entomopathogens with activity against plant pathogens: Ecology and evolution. BioControl.

[32] Jaber L.R. (2018). Seed inoculation with endophytic fungal entomopathogens promotes plant growth and reduces crown and root rot (CRR) caused by *Fusarium culmorum* in wheat. Planta.

[33] Fadiji A.E., Babalola O.O. (2020). Elucidating mechanisms of endophytes used in plant protection and other bioactivities with multifunctional prospects. Front. Bioeng. Biotechnol..

[34] Gao F.-K., Dai C.-C., Liu X.-Z. (2010). Mechanisms of fungal endophytes in plant protection against pathogens. Afr. J. Microbiol. Res..

[35] Joseph B., Priya R.M. (2011). Bioactive Compounds from Endophytes and their Potential in. Am. J. Biochem. Mol. Biol..

[36] Fadiji A.E., Babalola O.O. (2020). Exploring the potentialities of beneficial endophytes for improved plant growth. Saudi J. Biol. Sci..

[37] Pell J., Eilenberg J., Hajek A., Steinkraus D. (2001). Biology, ecology and pest management potential of Entomophthorales. Fungi Biocontrol Agents Prog. Probl. Potential.

[38] Shah P., Pell J. (2003). Entomopathogenic fungi as biological control agents. Appl. Microbiol. Biotechnol..

[39] Eilenberg J., Hajek A., Lomer C. (2001). Suggestions for unifying the terminology in biological control. BioControl.

[40] Samways M.J. (1981). Biological Control of Pests and Weeds.

[41] Hajek A.E., Elkinton J.S., Witcosky J.J. (1996). Introduction and spread of the fungal pathogen *Entomophaga*
*maimaiga* (Zygomycetes: Entomophthorales) along the leading edge of gypsy moth (Lepidoptera: Lymantriidae) spread. Environ. Entomol..

[42] Weiser J., Bucher G., Poinar G., Huffaker C.B., Messenger P.S. (1976). Host relationships and utility of pathogens. The Theory and Practice of Biological Control.

[43] Fuxa J.R. (1998). Environmental manipulation for microbial control of insects. Conservation Biological Control.

[44] Gurr G.M., Reynolds O.L., Johnson A.C., Desneux N., Zalucki M.P., Furlong M.J., Li Z., Akutse K.S., Chen J., Gao X. (2018). Landscape ecology and expanding range of biocontrol agent taxa enhance prospects for diamondback moth management. A review. Agron. Sustain. Dev..

[45] Montemayor C.O., Avery P.B., Cave R.D. (2016). Infection and mortality of *Microtheca*
*ochroloma* (Coleoptera: Chrysomelidae) by *Isaria*
*fumosorosea* (Hypocreales: Cordycipitaceae) under laboratory conditions. Biocontrol Sci. Technol..

[46] Goettel M.S., Poprawski T., Vandenberg J., Li Z., Roberts D.W., Laird M., Lacey L.A., Davidson E.W. (1990). Safety to nontarget invertebrates of fungal biocontrol agents. Safety of Microbial Insecticides.

[47] Bidochka M.J., Small C.L., Vega F.E., Blackwell M. (2005). Phylogeography of *Metarhizium*, an insect pathogenic fungus. Insect-Fungal Associations.

[48] Ekesi S., Maniania N., Onu I., Löhr B. (1998). Pathogenicity of entomopathogenic fungi (Hyphomycetes) to the legume flower thrips, *Megalurothrips sjostedti* (Trybom) (Thysan., Thripidae). J. Appl. Entomol..

[49] Shen F.-T., Yen J.-H., Liao C.-S., Chen W.-C., Chao Y.-T. (2019). Screening of Rice Endophytic Biofertilizers with Fungicide Tolerance and Plant Growth-Promoting Characteristics. Sustainability.

[50] Khan A.L., Waqas M., Kang S.-M., Al-Harrasi A., Hussain J., Al-Rawahi A., Al-Khiziri S., Ullah I., Ali L., Jung H.-Y. (2014). Bacterial Endophyte *Sphingomonas* sp LK11 Produces Gibberellins and IAA and Promotes Tomato Plant Growth. J. Microbiol..

[51] Rajamanikyam M., Vadlapudi V., Upadhyayula S.M. (2017). Endophytic fungi as novel resources of natural therapeutics. Braz. Arch. Biol. Technol..

[52] Gibson D.M., Donzelli B.G., Krasnoff S.B., Keyhani N.O. (2014). Discovering the secondary metabolite potential encoded within entomopathogenic fungi. Nat. Prod. Rep..

[53] Hardoim P.R., Van Overbeek L.S., Berg G., Pirttilä A.M., Compant S., Campisano A., Döring M., Sessitsch A. (2015). The hidden world within plants: Ecological and evolutionary considerations for defining functioning of microbial endophytes. Microbiol. Mol. Biol. Rev..

[54] Dara S.K. (2019). Non-Entomopathogenic Roles of Entomopathogenic Fungi in Promoting Plant Health and Growth. Insects.

[55] Johnston-Monje D., Raizada M.N. (2011). Conservation and diversity of seed associated endophytes in Zea across boundaries of evolution, ethnography and ecology. PLoS ONE.

[56] De Faria M.R., Wraight S.P. (2007). Mycoinsecticides and mycoacaricides: A comprehensive list with worldwide coverage and international classification of formulation types. Biol. Control.

[57] Wraight S., Jackson M., De Kock S., Butt T.M., Jackson C., Magan N. (2001). Production, stabilization and formulation of fungal biocontrol agents. Fungi as Biocontrol Agents: Progress, Problems and Potential.

[58] Gray S.N., Markham P. (1997). A model to explain the growth kinetics of the aphid-pathogenic fungus *Erynia neoaphidis* in liquid culture. Mycol. Res..

[59] Raya-Díaz S., Sánchez-Rodríguez A.R., Segura-Fernández J.M., Del Campillo M.D.C., Quesada-Moraga E. (2017). Entomopathogenic fungi-based mechanisms for improved Fe nutrition in sorghum plants grown on calcareous substrates. PLoS ONE.

[60] Zimmermann G. (2007). Review on safety of the entomopathogenic fungi *Beauveria bassiana* and *Beauveria brongniartii*. Biocontrol Sci. Technol..

[61] Maina U., Galadima I., Gambo F., Zakaria D. (2018). A review on the use of entomopathogenic fungi in the management of insect pests of field crops. J. Entomol. Zool. Stud..

[62] Skinner M., Parker B.L., Kim J.S., Abrol D.P. (2014). Role of entomopathogenic fungi in integrated pest management. Integrated Pest Management.

[63] Keller S., Zimmermann G., Wilding N., Collins N.M., Hammond P.M., Weber J.F. (1989). Mycopathogens of soil insects. Insect-Fungus Interact.

[64] Vestergaard S., Cherry A., Keller S., Goettel M. (2003). Safety of hyphomycete fungi as microbial control agents. Environmental Impacts of Microbial Insecticides.

[65] Zimmermann G. (1998). Suggestions for a standardized method for reisolation of entomopathogenic fungi from soil using the bait method. IOBC WPRS Bull..

[66] Meyling N.V. (2007). Methods for Isolation of Entomopathogenic Fungi from the Soil Environment.

[67] Pérez-González V.H., Guzmán-Franco A.W., Alatorre-Rosas R., Hernández-López J., Hernández-López A., Carrillo-Benítez M.G., Baverstock J. (2014). Specific diversity of the entomopathogenic fungi *Beauveria* and *Metarhizium* in Mexican agricultural soils. J. Invertebr. Pathol..

[68] Muñiz-Reyes E., Guzmán-Franco A., Sánchez-Escudero J., Nieto-Angel R. (2014). Occurrence of entomopathogenic fungi in tejocote (*Crataegus mexicana*) orchard soils and their pathogenicity against *Rhagoletis pomonella*. J. Appl. Microbiol..

[69] Cabrera-Mora J., Guzmán-Franco A., Santillán-Galicia M., Tamayo-Mejía F. (2019). Niche separation of species of entomopathogenic fungi within the genera *Metarhizium* and *Beauveria* in different cropping systems in Mexico. Fungal Ecol..

[70] Plantey R.L., Papura D., Couture C., Thiéry D., Pizzuolo P.H., Bertoldi M.V., Lucero G.S. (2019). Characterization of entomopathogenic fungi from vineyards in Argentina with potential as biological control agents against the European grapevine moth Lobesia botrana. BioControl.

[71] Goettel M.S., Inglis G.D. (1997). Fungi: Hyphomycetes. Manual of Techniques in Insect Pathology.

[72] Hu G., St Leger J. (2002). Field studies using a recombinant mycoinsecticide (*Metarhizium anisopliae*) reveal that it is rhizosphere competent. Appl. Environ. Microbiol..

[73] Dong T., Zhang B., Jiang Y., Hu Q. (2016). Isolation and classification of fungal whitefly entomopathogens from soils of Qinghai-Tibet Plateau and Gansu Corridor in China. PLoS ONE.

[74] Orgiazzi A., Dunbar M.B., Panagos P., de Groot G.A., Lemanceau P. (2015). Soil biodiversity and DNA barcodes: Opportunities and challenges. Soil Biol. Biochem..

[75] Gathage J.W., Lagat Z.O., Fiaboe K.K.M., Akutse K.S., Ekesi S., Maniania N.K. (2016). Prospects of fungal endophytes in the control of *Liriomyza leafminer* flies in common bean *Phaseolus vulgaris* under field conditions. BioControl.

[76] Canassa F., Esteca F.C., Moral R.A., Meyling N.V., Klingen I., Delalibera I. (2020). Root inoculation of strawberry with the entomopathogenic fungi *Metarhizium robertsii* and *Beauveria bassiana* reduces incidence of the twospotted spider mite and selected insect pests and plant diseases in the field. J. Pest Sci..

[77] Mantzoukas S., Chondrogiannis C., Grammatikopoulos G. (2015). Effects of three endophytic entomopathogens on sweet sorghum and on the larvae of the stalk borer *S**esamia nonagrioides*. Entomol. Exp. Appl..

[78] Cherry A.J., Banito A., Djegui D., Lomer C. (2004). Suppression of the stem-borer *Sesamia calamistis* (Lepidoptera; Noctuidae) in maize following seed dressing, topical application and stem injection with African isolates of *Beauveria bassiana*. Int. J. Pest Manag..

[79] Schaerffenberg B. (1968). Untersuchungen über die Wirkung der Insektentötenden Pilze *Beauveria bassiana* (Bals. Vuill.) und *Metarrhizium anisopliae* (Metsch.) Sorok. auf Warmblütler. Entomophaga.

[80] Goettel M.S., Jaronski S.T. (1997). Safety and registration of microbial agents for control of grasshoppers and locusts. Mem. Entomol. Soc. Can..

[81] Jing T., Li L., Chi H., Ruiyan M. (2013). The study on the acute toxicity and dermal sensitization of *Isaria fumosorosea*. J. Shanxi Agric. Univ. (Nat. Sci. Ed.).

[82] Shadduck J.A., Roberts D.W., Lause S. (1982). Mammalian safety tests of *Metarhizium anosipliae*: Preliminary results. Environ. Entomol..

[83] Siegel J., Shadduck J., Laird M., Lacey L.A., Davidson E.W. (1990). Safety of microbial insecticides to vertebrates—Humans. Safety of Microbial Insecticides.

[84] Fan M., Guo C., Li N. (1990). Application of Metarhizium Anisopliae against Forest Pests.

[85] Jevanand H., Kannan N. (1995). Evaluation of *Metarhizium anisopliae* as a biocontrol agent for coconut pest *Oryctes rhinoceros* and its mammalian toxicity test on rats. J. Ecotoxicol. Environ. Monit..

[86] Toriello C., Pérez-Torres A., Burciaga-Díaz A., Navarro-Barranco H., Pérez-Mejía A., Lorenzana-Jimenez M., Mier T. (2006). Lack of acute pathogenicity and toxicity in mice of an isolate of *Metarhizium anisopliae* var. anisopliae from spittlebugs. Ecotoxicol. Environ. Saf..

[87] Sachs S.W., Baum J., Mies C. (1985). *Beauvaria bassiana* keratitis. Br. J. Ophthalmol..

[88] Low C.D., Badenoch P.R., Coster D.J. (1997). *Beauveria bassiana* keratitis cured by deep lamellar dissection. Cornea.

[89] Kisla T.A., Cu-Unjieng A., Sigler L., Sugar J. (2000). Medical management of *Beauveria bassiana* keratitis. Cornea.

[90] Sigler L., Howard D.H. (2002). Miscellaneous opportunistic fungi: Microascaceae and other Ascomycetes, Hyphomycetes, Coelomycetes, and Basidiomycetes. Pathogenic Fungi in Humans and Animals.

[91] Clay K. (1988). Fungal endophytes of grasses: A defensive mutualism between plants and fungi. Ecology.

[92] Hoveland C.S. (1993). Importance and economic significance of the *Acremonium* endophytes to performance of animals and grass plant. Agric. Ecosyst. Environ..

[93] Latch G. (1976). Studies on the susceptibility of *Oryctes rhinoceros* to some entomogenous fungi. Entomophaga.

[94] Hartfield C., Campbell C., Hardie J., Pickett J., Wadhams L. (2001). Pheromone traps for the dissemination of an entomopathogen by the damson-hop aphid *Phorodon humuli*. Biocontrol Sci. Technol..

[95] Pell J., Macaulay E., Wilding N. (1993). A pheromone trap for dispersal of the pathogen *Zoophthora radicans* Brefeld.(Zygomycetes: Entomophthorales) amongst populations of the diamondback moth, *Plutella xylostella* L.(Lepidoptera: Yponomeutidae). Biocontrol Sci. Technol..

[96] Canassa F., Tall S., Moral R.A., de Lara I.A., Delalibera I., Meyling N.V. (2019). Effects of bean seed treatment by the entomopathogenic fungi *Metarhizium robertsii* and *Beauveria bassiana* on plant growth, spider mite populations and behavior of predatory mites. Biol. Control.

[97] Jaber L.R., Araj S.-E. (2018). Interactions among endophytic fungal entomopathogens (Ascomycota: Hypocreales), the green peach aphid *Myzus persicae* Sulzer (Homoptera: Aphididae), and the aphid endoparasitoid *Aphidius colemani* Viereck (Hymenoptera: Braconidae). Biol. Control.

[98] Akutse K.S., Fiaboe K.K., Van den Berg J., Ekesi S., Maniania N.K. (2014). Effects of endophyte colonization of *Vicia faba* (Fabaceae) plants on the life–history of leafminer parasitoids *Phaedrotoma scabriventris* (Hymenoptera: Braconidae) and *Diglyphus isaea* (Hymenoptera: Eulophidae). PLoS ONE.

[99] Tian J., Diao H.L., Liang L., Arthurs S., Hao C., Mascarin G.M., Ma R.Y. (2016). Host plants influence susceptibility of whitefly *Bemisia tabaci* (Hemiptera: Aleyrodidae) to the entomopathogenic fungus *Isaria fumosorosea* (Hypocreales: Cordycipitaceae). Biocontrol Sci. Technol..

[100] Cabanillas H.E., Jones W.A. (2013). Pathogenicity of *Isaria poprawskii* (Ascomycota: Hypocreales: Cordycipitaceae) against the glassy-winged sharpshooter, *Homalodisca vitripennis* (Hemiptera: Cicadellidae), under laboratory conditions. Crop Prot..

[101] Shapiro-Ilan D.I., Cottrell T.E., Jackson M.A., Wood B.W. (2008). Virulence of Hypocreales fungi to pecan aphids (Hemiptera: Aphididae) in the laboratory. J. Invertebr. Pathol..

[102] Conceschi M.R., D’Alessandro C.P., de Andrade Moral R., Demétrio C.G.B., Júnior I.D. (2016). Transmission potential of the entomopathogenic fungi *Isaria fumosorosea* and *Beauveria bassiana* from sporulated cadavers of *Diaphorina citri* and *Toxoptera citricida* to uninfected *D. citri* adults. BioControl.

[103] Tragust S., Mitteregger B., Barone V., Konrad M., Ugelvig L.V., Cremer S. (2013). Ants disinfect fungus-exposed brood by oral uptake and spread of their poison. Curr. Biol..

[104] Mburu D.M., Ochola L., Maniania N.K., Njagi P.G.N., Gitonga L.M., Ndung’u M.W., Wanjoya A.K., Hassanali A. (2009). Relationship between virulence and repellency of entomopathogenic isolates of *Metarhizium anisopliae* and *Beauveria bassiana* to the termite *Macrotermes michaelseni*. J. Insect Physiol..

[105] Meyling N.V., Pell J.K. (2006). Detection and avoidance of an entomopathogenic fungus by a generalist insect predator. Ecol. Entomol..

[106] Rännbäck L.-M., Cotes B., Anderson P., Rämert B., Meyling N.V. (2015). Mortality risk from entomopathogenic fungi affects oviposition behavior in the parasitoid wasp *Trybliographa rapae*. J. Invertebr. Pathol..

[107] Zimmermann G. (1984). Weitere Versuche mit *Metarhizium anisopliae* (Fungi imperfecti, Moniliales) zur Bekämpfung des Gefurchten Dickmaulrüßlers, Otiorhynchus sulcatus F., an Topfpflanzen im Gewächshaus. Bull. Rech. Agron. Gembloux.

[108] Inyang E., Butt T., Beckett A., Archer S. (1999). The effect of crucifer epicuticular waxes and leaf extracts on the germination and virulence of *Metarhizium anisopliae* conidia. Mycol. Res..

[109] Inyang E., Butt T., Doughty K., Todd A., Archer S. (1999). The effects of isothiocyanates on the growth of the entomopathogenic fungus *Metarhizium anisopliae* and its infection of the mustard beetle. Mycol. Res..

[110] Klingen I., Hajek A., Meadow R., Renwick J. (2002). Effect of brassicaceous plants on the survival and infectivity of insect pathogenic fungi. BioControl.

[111] Ramoska W., Todd T. (1985). Variation in efficacy and viability of *Beauveria bassiana* in the chinch bug (Hemiptera: Lygaeidae) as a result of feeding activity on selected host plants. Environ. Entomol..

[112] Poprawski T., Greenberg S., Ciomperlik M. (2000). Effect of host plant on *Beauveria bassiana*-and *Paecilomyces fumosoroseus*-induced mortality of *Trialeurodes vaporariorum* (Homoptera: Aleyrodidae). Environ. Entomol..

[113] Poprawski T.J., Jones W.J. (2001). Host plant effects on activity of the mitosporic fungi *Beauveria bassiana* and *Paecilomyces fumosoroseus* against two populations of *Bemisia* whiteflies (Homoptera: Aleyrodidae). Mycopathologia.

[114] Kouassi M., Coderre D., Todorova S.I. (2003). Effect of plant type on the persistence of *Beauveria bassiana*. Biocontrol Sci. Technol..

[115] Wraight S.P., Carruthers R.I., Jaronski S.T., Bradley C.A., Garza C.J., Galaini-Wraight S. (2000). Evaluation of the entomopathogenic fungi *Beauveria bassiana* and *Paecilomyces fumosoroseus* for microbial control of the silverleaf whitefly, *Bemisia argentifolii*. Biol. Control.

[116] Luz C., Fargues J., Romaña C. (2003). Influence of starvation and blood meal-induced moult on the susceptibility of nymphs of *Rhodnius prolixus* Stål (Hem., Triatominae) to *Beauveria bassiana* (Bals.) Vuill. infection. J. Appl. Entomol..

[117] Tang L.-C., Hou R. (2001). Effects of environmental factors on virulence of the entomopathogenic fungus, *Nomuraea rileyi*, against the corn earworm, *Helicoverpa armigera* (Lep., Noctuidae). J. Appl. Entomol..

[118] Walstad J., Anderson R., Stambaugh W. (1970). Effects of environmental conditions on two species of muscardine fungi (*Beauveria bassiana* and *Metarrhizium anisopliae*). J. Invertebr. Pathol..

[119] Milner R. (1997). Prospects for biopesticides for aphid control. Entomophaga.

[120] Bai Y., Cui Y., Cao N., Liu Y., Bugti G.A., Wang B. (2016). Effects of humidity and temperature on the pathogenecity of *Beauveria bassiana* against *Stephanitis nashi* and *Locusta migratoria* manilensis. Chin. J. Biol. Control.

[121] Dimbi S., Maniania N.K., Lux S.A., Mueke J.M. (2004). Effect of constant temperatures on germination, radial growth and virulence of *Metarhizium anisopliae* to three species of African tephritid fruit flies. BioControl.

[122] Lord J.C. (2005). Low humidity, moderate temperature, and desiccant dust favor efficacy of *Beauveria bassiana* (Hyphomycetes: Moniliales) for the lesser grain borer, *Rhyzopertha dominica* (Coleoptera: Bruchidae). Biol. Control.

[123] Moore D., Bridge P., Higgins P., Bateman R., Prior C. (1993). Ultra-violet radiation damage to *Metarhizium flavoviride* conidia and the protection given by vegetable and mineral oils and chemical sunscreens. Ann. Appl. Biol..

[124] Alves R.T., Bateman R.P., Prior C., Leather S.R. (1998). Effects of simulated solar radiation on conidial germination of *Metarhizium anisopliae* in different formulations. Crop Prot..

[125] Inyang E.N., McCartney H.A., Oyejola B., Ibrahim L., Archer S.A. (2000). Effect of formulation, application and rain on the persistence of the entomogenous fungus *Metarhizium anisopliae* on oilseed rape. Mycol. Res..

[126] Shah P.A., Douro-Kpindou O.-K., Sidibe A., Daffe C.O., Van der Pauuw H., Lomer C.J. (1998). Effects of the sunscreen oxybenzone on field efficacy and persistence of *Metarhizium flavoviride* conidia against *Kraussella amabile* (Orthoptera: Acrididae) in Mali, West Africa. Biocontrol Sci. Technol..

[127] Rangel D.E., Braga G.U., Flint S.D., Anderson A.J., Roberts D.W. (2004). Variations in UV-B tolerance and germination speed of *Metarhizium anisopliae* conidia produced on insects and artificial substrates. J. Invertebr. Pathol..

[128] Adeleke B.S., Babalola O.O. (2021). Biotechnological overview of agriculturally important endophytic fungi. Hortic. Environ. Biotechnol..

[129] Veen K., Ferron P. (1966). A selective medium for the isolation of *Beauveria*
*tenella* and of *Metarrhizium*
*anisopliae*. J. Invertebr. Pathol..

[130] Vestergaard S., Eilenberg J. Persistence of released *Metarhizium*
*anisopliae* in soil and prevalence in ground and rove beetles. Proceedings of the IOBC/WPRS Working Group: Insect Pathogens and Insect Parasitic Nematodes.

[131] Strasser H., Forer A., Schinner F., Jackson T.A., Glare T.R. (1996). Development of media for the selective isolation and maintenance of virulence of *Beauveria*
*brongniartii*. Microbial Control of Soil Dwelling Pests.

[132] Enkerli J., Widmer F., Keller S. (2004). Long-term field persistence of *Beauveria*
*brongniartii* strains applied as biocontrol agents against European cockchafer larvae in Switzerland. Biol. Control.

[133] Keller S., Kessler P., Schweizer C. (2003). Distribution of insect pathogenic soil fungi in Switzerland with special reference to *Beauveria*
*brongniartii* and *Metharhizium*
*anisopliae*. BioControl.

[134] Kessler P., Enkerl J., Schweize C., Keller S. (2004). Survival of *Beauveria*
*brongniartii* in the soil after application as a biocontrol agent against the European cockchafer *Melolontha*
*melolontha*. Biocontrol.

[135] Kessler P., Matzke H., Keller S. (2003). The effect of application time and soil factors on the occurrence of *Beauveria*
*brongniartii* applied as a biological control agent in soil. J. Invertebr. Pathol..

[136] Klingen I., Eilenberg J., Meadow R. (2002). Effects of farming system, field margins and bait insect on the occurrence of insect pathogenic fungi in soils. Agric. Ecosyst. Environ..

[137] Gao D., Tao Y. (2012). Current molecular biologic techniques for characterizing environmental microbial community. Front. Environ. Sci. Eng..

[138] Rungjindamai N., Pinruan U., Choeyklin R., Hattori T., Jones E. (2008). Molecular characterization of basidiomycetous endophytes isolated from leaves, rachis and petioles of the oil palm, *Elaeis*
*guineensis*. Thailand. Fungal Divers..

[139] Dissanayake A.J., Purahong W., Wubet T., Hyde K.D., Zhang W., Xu H., Zhang G., Fu C., Liu M., Xing Q. (2018). Direct comparison of culture-dependent and culture-independent molecular approaches reveal the diversity of fungal endophytic communities in stems of grapevine (*Vitis vinifera*). Fungal Divers..

[140] Mehmood A., Hussain A., Irshad M., Hamayun M., Iqbal A., Rahman H., Tawab A., Ahmad A., Ayaz S. (2019). Cinnamic acid as an inhibitor of growth, flavonoids exudation and endophytic fungus colonization in maize root. Plant Physiol. Biochem..

[141] Jaronski S.T., Ekesi S., Maniania N.K. (2007). Soil ecology of the entomopathogenic ascomycetes: A critical examination of what we (think) we know. Use of Entomopathogenic Fungi in Biological Pest Management.

[142] Bridge P., Spooner B. (2001). Soil fungi: Diversity and detection. Plant Soil.

[143] Onofre S.B., Miniuk C.M., de Barros N.M., Azevedo J.L. (2001). Pathogenicity of four strains of entomopathogenic fungi against the bovine tick *Boophilus microplus*. Am. J. Vet. Res..

[144] Sanjuan T.I., Franco-Molano A.E., Kepler R.M., Spatafora J.W., Tabima J., Vasco-Palacios A.M., Restrepo S. (2015). Five new species of entomopathogenic fungi from the Amazon and evolution of neotropical Ophiocordyceps. Fungal Biol..

[145] Wakil W., Ghazanfar M.U., Riasat T., Kwon Y.J., Qayyum M.A., Yasin M. (2013). Occurrence and diversity of entomopathogenic fungi in cultivated and uncultivated soils in Pakistan. Entomol. Res..

[146] Samson R., Evans H., Latge J. (1988). Atlas of Entomopathogenic Fungi.

[147] Evans H., Wilding N., Collins N.M., Hammond P.M., Weber J.F. (1989). Mycopathogens of insects of epigeal and aerial habitats. Insect-Fungus Interact.

[148] Eilenberg J. (2002). Biology of Fungi from the Order Entomophthorales with Emphasis on the Genera Entomophthora, Strongwellsea and Eryniopsis. A Contribution to Insect Pathology and Biological Control.

[149] Hawksworth D.L., Rossman A.Y. (1997). Where are all the undescribed fungi?. Phytopathology.

[150] Hawksworth D.L. (2001). The magnitude of fungal diversity: The 1· 5 million species estimate revisited. Mycol. Res..

[151] Fisher J.J., Rehner S.A., Bruck D.J. (2011). Diversity of rhizosphere associated entomopathogenic fungi of perennial herbs, shrubs and coniferous trees. J. Invertebr. Pathol..

[152] Li Z., Li X., Yang S. (2014). Edaphic factor that affect the activity of entomopathogenic fungus *Metarhizium anisopliae*. Biotechnol. Bull..

[153] Clerk G., Madelin M. (1965). The longevity of conidia of three insect-parasitizing hyphomycetes. Trans. Br. Mycol. Soc..

[154] Meyling N.V., Eilenberg J. (2006). Isolation and characterisation of *Beauveria bassiana* isolates from phylloplanes of hedgerow vegetation. Mycol. Res..

[155] Glare T.R., Milner R.J., Arora D.K., Ajello L., Mukerji K.G. (1991). Ecology of entomopathogenic fungi. Handbook of Applied Mycology, Humans, Animals, and Insects.

[156] Nielsen C., Hajek A.E., Humber R.A., Eilenberg J. (1998). Soil: A natural source of entomophthoralean fungi infecting aphids. Insect Pathog. Insect Parasit. Nematodes IOBC Bull..

[157] Feng M.-G., Johnson J.B., Halbert S.E. (1991). Natural control of cereal aphids (Homoptera: Aphididae) by entomopathogenic fungi (Zygomycetes: Entomophthorales) and parasitoids (Hymenoptera: Braconidae and Encyrtidae) on irrigated spring wheat in southwestern Idaho. Environ. Entomol..

[158] Feng M.-G., Nowierski R.M., Klein R.E., Scharen A.L., Sands D.C. (1992). Spherical hyphal bodies of *Pandora neoaphidis* (Remaudiere and Hennebert) Humber (Zygomycetes; Entomophthorales) on *Acyrthosiphon pisum* (Harris) (Homoptera: Aphididae): A potential overwintering form. Pan-Pac. Entomol..

[159] Compant S., Van Der Heijden M.G., Sessitsch A. (2010). Climate change effects on beneficial plant–microorganism interactions. FEMS Microbiol. Ecol..

[160] Van der Putten W.H. (2003). Plant defense belowground and spatiotemporal processes in natural vegetation. Ecology.

[161] Enebe M.C., Babalola O.O. (2018). The influence of plant growth-promoting rhizobacteria in plant tolerance to abiotic stress: A survival strategy. Appl. Microbiol. Biotechnol..

[162] Alves S., Marchini L., Pereira R., Baumgratz L. (1996). Effects of some insect pathogens on the Africanized honey bee, *Apis mellifera* L. (Hym., Apidae). J. Appl. Entomol..

[163] Copping L.G. (2004). The manual of biocontrol agents: British Crop Protection Council. Crop Prot..

[164] Butt T., Ibrahim L., Ball B., Clark S. (1994). Pathogenicity of the entomogenous fungi *Metarhizium anisopliae* and *Beauveria bassiana* against crucifer pests and the honey bee. Biocontrol Sci. Technol..

[165] Peveling R., Attignon S., Langewald Je Ouambama Z. (1999). An assessment of the impact of biological and chemical grasshopper control agents on ground-dwelling arthropods in Niger, based on presence/absence sampling. Crop Prot..

[166] Reynaldi F.J., Lucia M., Garcia M.L.G. (2015). *Ascosphaera apis*, the entomopathogenic fungus affecting larvae of native bees (*Xylocopa augusti*): First report in South America. Revista Iberoamericana de Micologia.

[167] Toledo-Hernández R., Ruíz-Toledo J., Toledo J., Sánchez D. (2016). Effect of three entomopathogenic fungi on three species of stingless bees (Hymenoptera: Apidae) under laboratory conditions. J. Econ. Entomol..

[168] Hokkanen H.M.T., Zeng Q.-Q., Menzler-Hokkanen I., Hokkanen H.M.T., Hajek A.E. (2003). Assessing the impacts of *Metarhizium* and *Beauveria* on bumblebees. Environmental Impacts of Microbial Insecticides.

[169] Wang J.-J., Yang L., Qiu X., Liu Y.-G., Zhou W., Wan Y.-J. (2013). Diversity analysis of *Beauveria bassiana* isolated from infected silkworm in southwest China based on molecular data and morphological features of colony. World J. Microbiol. Biotechnol..

[170] Danfa A., Van der Valk H. (1999). Laboratory testing of *Metarhizium* spp. and Beauveria bassiana on Sahelian non-target arthropods. Biocontrol. Sci. Technol..

[171] Nielsen C., Skovgård H., Steenberg T. (2005). Effect of *Metarhizium anisopliae* (Deuteromycotina: Hyphomycetes) on survival and reproduction of the filth fly parasitoid, *Spalangia cameroni* (Hymenoptera: Pteromalidae). Environ. Entomol..

[172] Weng Q., Zhang X., Chen W., Hu Q. (2019). Secondary metabolites and the risks of *Isaria fumosorosea* and *Isaria farinosa*. Molecules.

[173] Fan M., Li J., Guo C., Hashan E. (1991). Persistent forms and survival potential of *Metarhizium anisopliae* in soil. J. Northwest. Coll For..

[174] Li Y., Hu Z., Hu Z., Xiao H., Li H. (2018). Survival dynamics of *Beauveria bassiana* Bb09in the muscardine cadaver of *Hyphantria cunea* and surrounding soil. For. Ecol. Sci..

[175] Zhang Y., Wu X., Ye B., Wang H., Shu J. (2017). Inhibition effects of soil bacteria on conidium germination of *Metarhizium pingshaense*. Chin. J. Biol. Control.

[176] Wu X., Zhang Y., Wu P., Ye B., Wang H., Shu J. (2014). Influence of temperature, moisture, and soil type on conidium germination of *Metarhizium pingshaense* in soil. Chin. J. Biol. Control.

[177] Jaronski S.T., Lord J., Rosinska J., Bradley C., Hoelmer K., Simmons G., Osterlind R., Brown C., Staten R. (1998). Effect of a Beauveria Bassiana-Based Mycoinsecticide on Beneficial Insects Under Field Conditions.

[178] Goettel M.S., Hajek A.E., Siegel J.P., Evans H.C., Butt T.M., Jackson C., Magan N. (2001). Safety of fungal biocontrol agents. Fungi as Biocontrol Agents, Progress, Problems, and Potential.

[179] Jarvis B.B., Wells K.M., Lee Y.-W., Bean G.A., Kommedahl T., Barros C.S., Barros S.S. (1987). Macrocyclic trichothecene mycotoxins in Brazilian species of Baccharis. Phytopathology.

[180] Stuedemann J.A., Hoveland C.S. (1988). Fescue endophyte: History and impact on animal agriculture. J. Prod. Agric..

[181] Schmidt S., Osborn T. (1993). Effects of endophyte-infected tall fescue on animal performance. Agric. Ecosyst. Environ..

[182] Clay K. (1994). The Potential Role of Endophytes in Ecosystems.

[183] Moretti A., Belisario A., Tafuri A., Ritieni A., Corazza L., Logrieco A. (2002). Production of beauvericin by different races of *Fusarium oxysporum* f. sp. melonis, the Fusarium wilt agent of muskmelon. Eur. J. Plant Pathol..

[184] El-Kadi M., Xara L., De Matos P., Da Rocha J., De Oliveira D. (1983). Effects of the entomopathogen *Metarhizium anisopliae* on guinea pigs and mice. Environ. Entomol..

[185] Bailey V. (1903). Sleepy grass and its effect on horses. Science.

[186] Nobindro U. (1934). Grass poisoning among cattle and goats in Assam. Indian Vet. J..

[187] Tibbets T., Faeth S.H. (1999). Neotyphodium endophytes in grasses: Deterrents or promoters of herbivory by leaf-cutting ants?. Oecologia.

[188] Saikkonen K., Faeth S.H., Helander M., Sullivan T. (1998). Fungal endophytes: A continuum of interactions with host plants. Annu. Rev. Ecol. Syst..

[189] Schulz B., Römmert A.-K., Dammann U., Aust H.-J., Strack D. (1999). The endophyte-host interaction: A balanced antagonism?. Mycol. Res..

[190] Richardson M.D., Bacon C.W., White J.F. (2000). Alkaloids of endophyte-infected grasses: Defence chemicals or biological anomalies. Microbial Endophytes.

[191] Schulz B., Boyle C. (2005). The endophytic continuum. Mycol. Res..

[192] Clay K., Cheplick G.P., Marks S. (1989). Impact of the fungus *Balansia henningsiana* on *Panicum agrostoides*: Frequency of infection, plant growth and reproduction, and resistance to pests. Oecologia.

[193] Gehring C.A., Whitham T.G. (1994). Interactions between aboveground herbivores and the mycorrhizal mutualists of plants. Trends Ecol. Evol..

[194] Fitter A., Garbaye J. (1994). Interactions between mycorrhizal fungi and other soil organisms. Plant Soil.

[195] Mueller R.C., Sthultz C.M., Martinez T., Gehring C.A., Whitham T.G. (2005). The relationship between stem-galling wasps and mycorrhizal colonization of *Quercus turbinella*. Botany.

[196] Gange A.C., Brown V.K., Aplin D.M. (2005). Ecological specificity of arbuscular mycorrhizae: Evidence from foliar-and seed-feeding insects. Ecology.

[197] Stenzel K. (1992). Mode of action and spectrum of activity of BIO 1020 (*Metarhizium anisopliae*). Pflanzenschutz-Nachr. Bayer.

[198] Claeson A.-S., Levin J.-O., Blomquist G., Sunesson A.-L. (2002). Volatile metabolites from microorganisms grown on humid building materials and synthetic media. J. Environ. Monit..

[199] Matysik S., Herbarth O., Mueller A. (2009). Determination of microbial volatile organic compounds (MVOCs) by passive sampling onto charcoal sorbents. Chemosphere.

[200] Martin P.A., Schroder R.F. (2000). The effect of cucurbitacin E glycoside, a feeding stimulant for corn rootworm, on biocontrol fungi: *Beauveria bassiana* and *Metarhizium anisopliae*. Biocontrol Sci. Technol..

[201] Van Overbeek L., Van Elsas J.D. (2008). Effects of plant genotype and growth stage on the structure of bacterial communities associated with potato (*Solanum tuberosum* L.). FEMS Microbiol. Ecol..

[202] Sánchez-Rodríguez A.R., Raya-Díaz S., Zamarreño Á.M., García-Mina J.M., del Campillo M.C., Quesada-Moraga E. (2018). An endophytic *Beauveria bassiana* strain increases spike production in bread and durum wheat plants and effectively controls cotton leafworm (*Spodoptera littoralis*) larvae. Biol. Control.

[203] Andreozzi A., Prieto P., Mercado-Blanco J., Monaco S., Zampieri E., Romano S., Valè G., Defez R., Bianco C. (2019). Efficient colonization of the endophytes *Herbaspirillum huttiense* RCA24 and *Enterobacter cloacae* RCA25 influences the physiological parameters of *Oryza sativa* L. cv. Baldo rice. Environ. Microbiol..

[204] Kandel S.L., Joubert P.M., Doty S.L. (2017). Bacterial endophyte colonization and distribution within plants. Microorganisms.

[205] Bamisile B.S., Akutse K.S., Dash C.K., Qasim M., Ramos Aguila L.C., Ashraf H.J., Huang W., Hussain M., Chen S., Wang L. (2020). Effects of seedling age on colonization patterns of *Citrus limon* plants by endophytic *Beauveria bassiana* and *Metarhizium anisopliae* and their influence on seedlings growth. J. Fungi.

[206] Faria M., Wraight S.P. (2001). Biological control of *Bemisia tabaci* with fungi. Crop Prot..

[207] Cuthbertson A.G., Blackburn L.F., Eyre D.P., Cannon R.J., Miller J., Northing P. (2011). *Bemisia tabaci*: The current situation in the UK and the prospect of developing strategies for eradication using entomopathogens. Insect Sci..

[208] Vega F.E., Dowd P.F., Lacey L.A., Pell J.K., Jackson D.M., Klein M.G., Lacey L.A., Kaya H.K. (2000). Dissemination of beneficial microbial agents by insects. Field Manual of Techniques in Invertebrate Pathology.

[209] Vandenberg J.D., Shelton A.M., Wilsey W.T., Ramos M. (1998). Assessment of *Beauveria bassiana* sprays for control of diamondback moth (Lepidoptera: Plutellidae) on crucifers. J. Econ. Entomol..

[210] Keller S. (1992). The Beauveria-Melolontha Project: Experiences with Regard to Locust and Grasshopper Control.

[211] Keller S., Schweizer C., Keller E., Brenner H. (1997). Control of white grubs (*Melolontha melolontha* L.) by treating adults with the fungus *Beauveria brongniartii*. Biocontrol Sci. Technol..

[212] Hartley S.E., Gange A.C. (2009). Impacts of plant symbiotic fungi on insect herbivores: Mutualism in a multitrophic context. Annu. Rev. Entomol..

[213] Zheng S.-J., Dicke M. (2008). Ecological genomics of plant-insect interactions: From gene to community. Plant. Physiol..

[214] Dong Y., Friedrich M. (2005). Nymphal RNAi: Systemic RNAi mediated gene knockdown in juvenile grasshopper. BMC Biotechnol..

[215] Zhou X., Wheeler M.M., Oi F.M., Scharf M.E. (2008). RNA interference in the termite *Reticulitermes flavipes* through ingestion of double-stranded RNA. Insect Biochem. Mol. Biol..

[216] Tian H., Peng H., Yao Q., Chen H., Xie Q., Tang B., Zhang W. (2009). Developmental control of a lepidopteran pest *Spodoptera exigua* by ingestion of bacteria expressing dsRNA of a non-midgut gene. PLoS ONE.

[217] Chen J., Zhang D., Yao Q., Zhang J., Dong X., Tian H., Chen J., Zhang W. (2010). Feeding-based RNA interference of a trehalose phosphate synthase gene in the brown planthopper, *Nilaparvata lugens*. Insect Mol. Biol..

[218] Huvenne H., Smagghe G. (2010). Mechanisms of dsRNA uptake in insects and potential of RNAi for pest control: A review. J. Insect Physiol..

[219] Wang F., Nong X., Hao K., Cai N., Wang G., Liu S., Ullah H., Zhang Z. (2020). Identification of the key genes involved in the regulation of symbiotic pathways induced by *Metarhizium anisopliae* in peanut (*Arachis hypogaea*) roots. 3 Biotech.

[220] Brader G., Compant S., Vescio K., Mitter B., Trognitz F., Ma L.-J., Sessitsch A. (2017). Ecology and genomic insights into plant-pathogenic and plant-nonpathogenic endophytes. Annu. Rev. Phytopathol..

[221] Fontana A., Reichelt M., Hempel S., Gershenzon J., Unsicker S.B. (2009). The effects of arbuscular mycorrhizal fungi on direct and indirect defense metabolites of *Plantago lanceolata* L.. J. Chem. Ecol..

[222] Al-Ani L.K.T., Aguilar-Marcelino L., Salazar-Vidal V.E., Becerra A.G., Raza W., Yadav A.N. (2021). Role of Useful Fungi in Agriculture Sustainability. Recent Trends in Mycological Research.

